# Mesenchymal Chondrosarcoma of the Mandible, a Big Dilemma: Report of a Rare Case in Mesenchymal Chondrosarcoma of the Mandible—Report of a Case With Discussion of Diagnostic and Therapeutic Dilemmas

**DOI:** 10.1155/2024/8884697

**Published:** 2024-10-28

**Authors:** Abbas Karimi, Samira Derakhshan, Farzaneh Mosavat, Zahra Gholamhoseini, Monir Moradzadeh Khiavi

**Affiliations:** ^1^Oral and Maxillofacial Surgery Department, Craniomaxillofacial Research Center, Shariati Hospital, Tehran University of Medical Sciences, Tehran, Iran; ^2^Oral and Maxillofacial Pathology Department, School of Dentistry, Tehran University of Medical Sciences, Tehran, Iran; ^3^Cancer Preclinical Imaging Group, Preclinical Core Facility, Tehran University of Medical Sciences, Tehran, Iran; ^4^Department of Oral & Maxillofacial Radiology, School of Dentistry, Tehran University of Medical Sciences, Tehran, Iran

## Abstract

Chondrosarcomas are a group of malignant neoplasms with cartilaginous matrix production mostly found in flat and peripheral long bones. Mesenchymal chondrosarcoma is one of the most unusual and rare histological variants of chondrosarcoma, with a distinct histopathological appearance and biologically aggressive behavior. The amount of cartilage in mesenchymal chondrosarcoma may be so abundant that it is easily found in random sections or so scarce that numerous sections are required to discover it. In such cases, it is tough to make an accurate diagnosis, which leads to a big dilemma for pathologists and surgeons regarding diagnosis and treatment. Here, we report a mandibular mesenchymal chondrosarcoma in a 38-year-old male with a diagnosis of malignant small round cell tumor in incisional biopsy without any bone or chondroid formation. After ruling out lymphoma, a complete lesion excision was done. Diagnosis of mesenchymal chondrosarcoma was confirmed with small foci of chondroid material and strong positivity of tumoral cells for CD99 and S100. We highlight the fact that incisional biopsy frequently fails to provide sufficient tissue to establish the diagnosis of mesenchymal chondrosarcoma. Adequate tissue with multiple sections, detailed histopathological examination, and adjunctive IHC study are the keys to a definitive diagnosis.

## 1. Introduction

Chondrosarcomas are a group of malignant neoplasms that produce a cartilaginous matrix. They are primarily found in flat and peripheral long bones. Following myeloma and osteosarcoma, chondrosarcoma is the third most prevalent bone malignancy, composing approximately 10%–20% of malignant bone tumors [1–3].

It rarely affects the head and neck region, which accounts for 0.1% of head and neck neoplasms. In the head and neck area, mostly chondrosarcomas arise from the maxilla rather than the mandible [1, 3, 4]. The preferred site in the mandible is the molar region, and they infrequently occur in the ramus, condyle, coronoid process, or symphysis [4, 5].

The literature describes several histological variants of chondrosarcomas: conventional (Grade I, Grade II, and Grade III), mesenchymal, dedifferentiated, clear cell, and myxoid [2].

Mesenchymal chondrosarcoma (MCS) was first described in 1959 [2, 6–8]. It is one of the most unusual and rare histological variants of chondrosarcoma with distinct histopathological appearance and biological aggressive behavior [6, 8, 9]. MCSs are rarely reported in the literature because of their rare incidence. However, the maxilla and mandible are the most commonly affected regions within the head and neck, though other bones are also involved. The maxilla is more affected than the mandible. In the mandible, the most common site is the premolar–molar region. However, other areas, such as the symphysis, coronoid, and condylar processes, may also be affected [10].

Histologically, MCS is seen with sheets or clusters of highly undifferentiated small ovoid cells intermixed with small zones of neoplastic cartilage [8]. The scarcity of cartilaginous tissue makes it more similar to various small-cell neoplasms that must be considered for the differential diagnosis, thus presenting a challenge [11].

This tumor accounts for 1%–10% of all chondrosarcomas and has a high propensity for the head and neck region. Unlike conventional chondrosarcomas, MCS is mainly seen in younger adults and is diagnosed at younger ages than other variants. The mean age of diagnosis is around 25–30 years [2, 7, 8]. It slightly occurs more in females, whereas classical chondrosarcomas prefer males [6, 8, 9].

MCS is also well known for its aggressive nature, late recurrence, and potential for distant metastasis. These tumors show local aggressive behavior as well as a high metastatic and recurrence potential [11].

Due to these features, the long-term prognosis is extremely poor. Considering the propensity of these tumors to metastasize and the poor prognosis of patients with MC, early identification may allow earlier more aggressive interventions. This underscores the importance of early and accurate diagnosis, as timely intervention can significantly impact patient outcomes.

This article reviews the literature on this lesion's clinical and pathological aspects. It also introduces a case of MCS of the mandible, focusing on its histological and radiological features and treatment.

## 2. Case

A 38-year-old male patient visited the oral and maxillofacial surgery department of Tehran University of Medical Sciences (TUMS) with the chief complaint of painless swelling in the right side of the lower jaw for 2 months.

Intraoral examination revealed an expansile lesion in the area of the right mandibular ramus with a firm consistency. There was no ulceration on the superficial mucosa.

**Figure 1:**
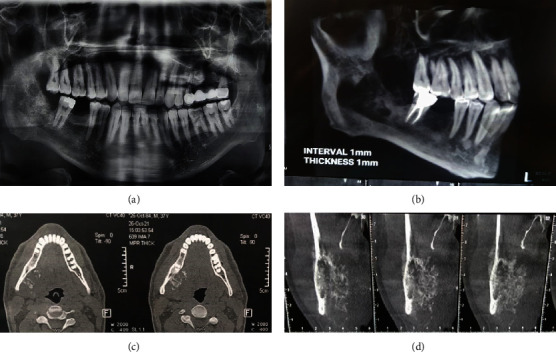
(a) Panoramic view. An ill-defined radiopaque lesion is seen on the right side of the mandible with an extension from the middle of the mandibular body to the ascending ramus. (b) 3D reconstruction. (c) An axial CT scan shows an ill-defined radiopaque mass arising from the medial aspect of the right mandibular body. (d) Coronal CT scan shows periosteal reaction on the medial and lateral aspects of the mandible, known as the Codman triangle and sunray pattern, respectively.

Radiographic examinations revealed an ill-defined destructive mixed radiolucent–radiopaque mass lesion located at the right mandibular ramus from the second molar to the sigmoid notch and from the alveolar crest to the inferior border of the mandible. The internal structure shows irregular osseous formation causing internal sunray appearance alongside Codman triangle periosteal reaction and PDL widening of the second molar. The lesion has also perforated the buccal and lingual cortex and extended to the soft tissue (Figures 1(a), 1(b), 1(c), and 1(d)).

**Figure 2:**
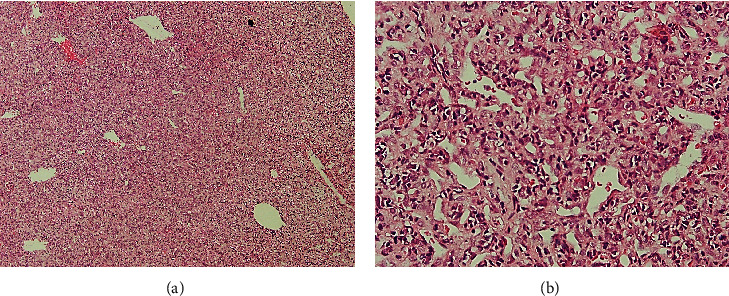
(a) Histopathologic evaluation. Sheets of small spindle to round undifferentiated cells (×100 magnification). (b) Histopathologic evaluations. High vascular background with staghorn (slit-like) capillaries (×400 magnification).

An incisional biopsy was taken from the oral mass under local anesthesia. The histopathological examination revealed a highly cellular neoplasm composed of sheets of small round to spindle cell proliferation, which shows a highly vascular network demonstrating rudimentary lumen formation and slit-like vessels (Figures 2(a) and 2(b)). Numerous atypical mitotic figures and necrosis were seen among tumoral cells. The proliferation of tumoral cells was evident between the regional bone trabeculae and bone marrow spaces. No bone or chondroid material was obvious. The differential diagnosis is composed of a large spectrum of small round cell tumors, including undifferentiated carcinoma, malignant melanoma, lymphoma, rhabdomyosarcoma, neuroblastoma, Ewing sarcoma, small-cell osteosarcoma, and MCS. An immunohistochemistry (IHC) study was done to make a definitive diagnosis.

The IHC study revealed that LCA, panCK, HNB45, MyoD1, and MALT1 were all negative in the tumoral cells, ruling out lymphoma, malignant melanoma, and undifferentiated carcinoma. However, positive scattered tumoral cells for CD99 and S100 indicated a possible diagnosis of MCS. The positivity of CD34 in stromal blood vessels further supported this diagnosis.

**Figure 3:**
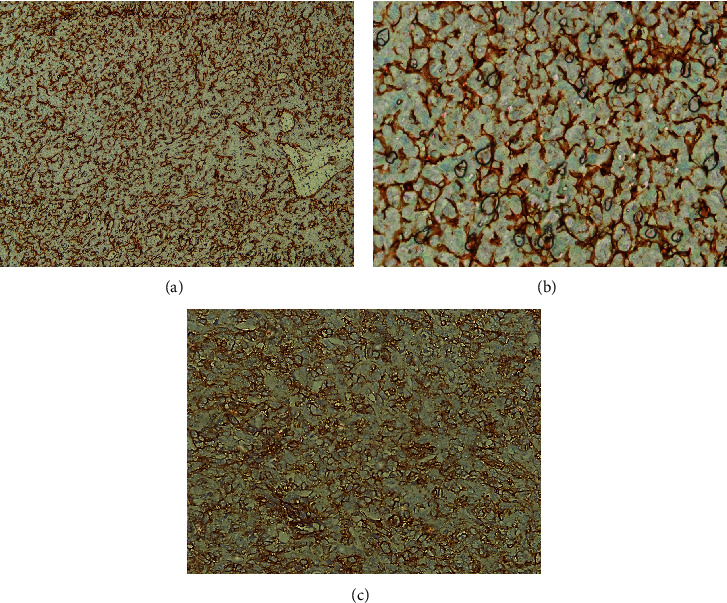
(a) IHC study. Positive immunoreaction for CD34 (×100 magnification). (b) IHC study. Positive immunoreactions for CD31 in highly vascular stroma (×400 magnification). (c) IHC study. Positive immunoreactions in membranous tumoral cells for CD99 (×400 magnification).

Based on IHC studies, we could rule out lymphoma, malignant melanoma, and undifferentiated carcinoma, but we could not rule out Ewing sarcoma and Ewing-like sarcomas (Figures 3(a), 3(b), and 3(c)). Since surgical treatment is the first treatment plan for all of these sarcomas and bone/cartilage-forming sarcomas, the surgeon decided to excise the whole lesion with safe margins after consultation in the hospital tumor board discussion meeting.

**Figure 4:**
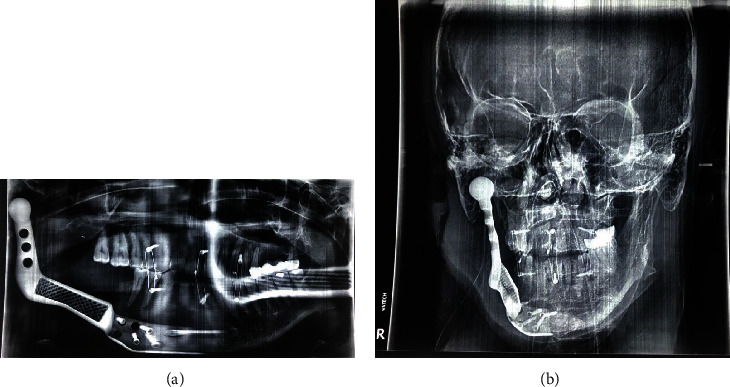
(a) Panoramic view immediately after tumor removal and placement of the prosthesis. (b) PA skull immediately after tumor removal and placement of the prosthesis.

Eventually, the patient underwent hemimandibulectomy. An extraoral incision was made from the right mastoid region to the midline to access the tumor and perform a partial mandibulectomy. The cutting area of the mandible was in front of the distal second premolar, with a 1.5-cm safe margin which is clear in Figure 4(a). According to all the guidelines, during the removal of all malignant tumors, including sarcomas, the frozen section was performed to confirm clearance of margins.

Based on paraclinical investigations, the submandibular gland and regional lymph nodes were free of any involvement and therefore were preserved.

**Figure 5:**
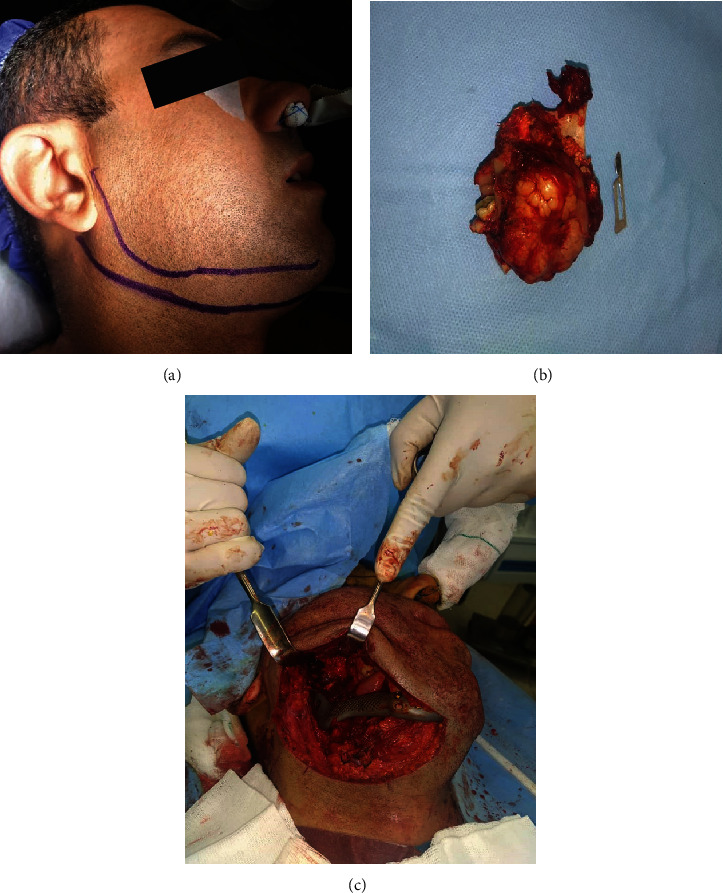
(a) Surgical treatment. Extraoral approach for removing the tumor. (b) Excision of the whole tumor with safe margins. (c) Prosthesis placement. The placement of the prosthesis provides continuity and some function for the mandible.

Following tumor resection, he received a costume-made titanium prosthesis to provide continuity of the mandible in the resection area (Figures 5(a), 5(b), and 5(c)).

Radiographs were made immediately after surgery for further investigations (Figures 4(a) and 4(b)).

**Figure 6:**
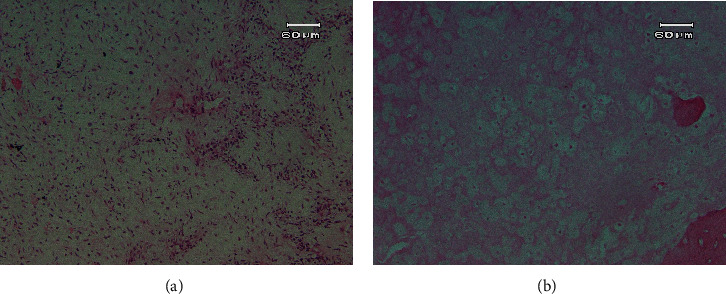
(a) Chondroid structure. Fibromyxoid tissue with areas mimicking chondroid structures (×100 magnification). (b) Chondroid structure. Chondrocytic lacunas in chondroid tissue with centrally located nuclei in some of them (×400 magnification).

Final histopathological examinations showed a biphasic neoplasm with undifferentiated round to spindle-shaped cells, nuclear pleomorphism, and some chondroid material foci. Staghorn vessels alongside frequent mitotic figures and small foci of necrosis were also observed (Figures 6(a) and 6(b)).

The tumor cells were strongly positive for CD99 (undifferentiated cells), and some cells with cartilage differentiation were positive for S100. Histologic findings and IHC results were consistent with MCS.

**Figure 7:**
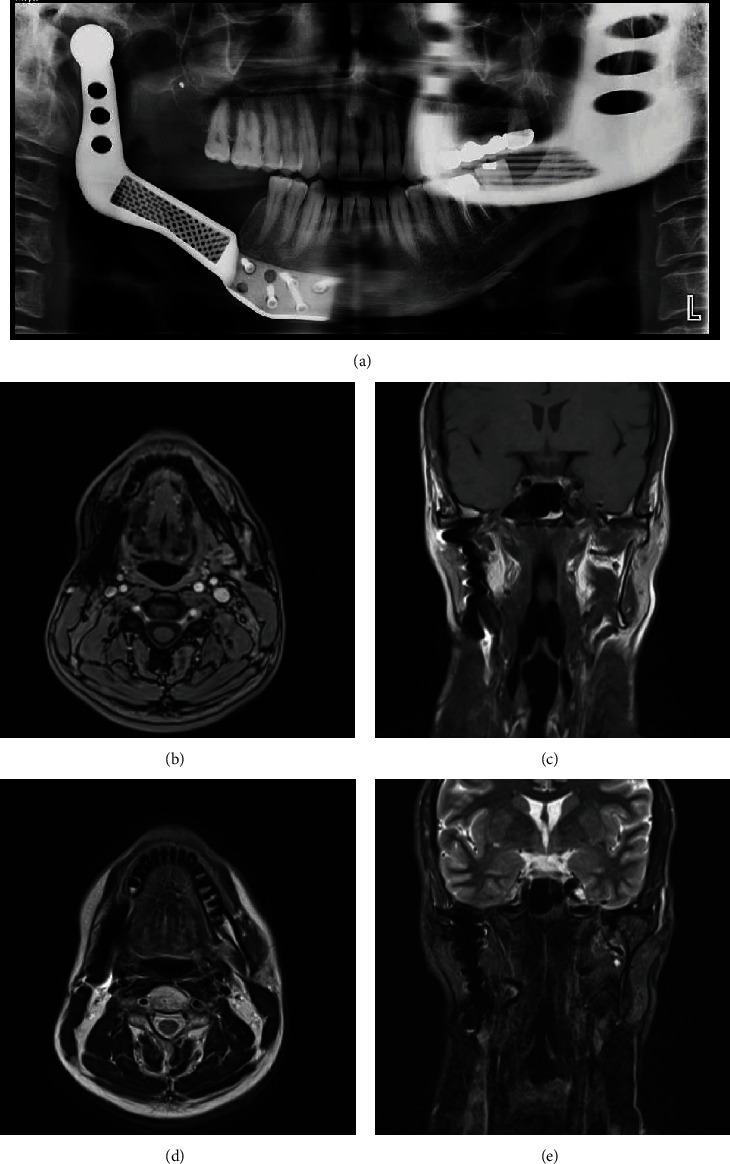
(a) Panoramic view 2 years after tumor removal. Evidence of cortication and healing is noted in the alveolar bone in the surgical bed. (b) MR images 2 years after treatment. (b, c) T1 postcontrast axial and coronal views. (d, e) T2 axial and coronal views. Evidence of previous surgery and blooming artifacts due to the prosthesis in the anatomic site of the right mandible is seen. As could be evaluated in the surgery bed, no abnormal enhancing lesion or any sign of recurrence is visualized.

The patient has been followed up every 6 months for 2 years. Chemotherapy and radiotherapy have been performed on the patient. He is in a good situation under routine follow-up visits without any evidence of recurrence (Figures 7(a), 7(b), 7(c), 7(d), and 7(e)).

## 3. Discussion

MCSs are rarely reported in the literature because of their rare incidence. However, the maxilla and mandible are the most commonly affected regions within the head and neck region, though other bones are also involved. When MCSs involve the jaws, the maxilla is more affected than the mandible. In the mandible, the most common site is the premolar–molar region. However, other regions, such as the symphysis, coronoid, and condylar processes, may also be affected [3, 12–14]. Stanbouly et al. conducted a literature review of 42 cases of MCS of the jaw from 1960 to 2021 [2]. We also reviewed 45 cases of mandibular MCS, as summarized in Table 1.

Diagnostic imaging is essential to the diagnostic workup, especially when evaluating the tumor size. Several radiological modalities are beneficial [45]. Conventional radiography is often the first modality performed during events, though no classical radiographic features exist for MCSs [1, 45]. Since it is a slow-growing tumor, its radiologic signs may be misleading and benign. However, it usually exhibits features compatible with malignancy in MDCT and CBCT [10]. These lesions frequently appear with ill-defined or ragged borders like other malignant tumors. The internal structure and radiographic appearance vary from completely radiolucent to mixed radiolucent–radiopaque based on the amount of calcified foci corresponding to the calcification of the neoplastic cartilaginous tissue [2, 10, 12, 25].

Shahidi et al. also reported ultrasonography (US) findings of chondrosarcoma of the mandible. The internal content, periphery, outline, extent, and new bone formation were characteristics detected on US images as precisely as on CT images. In addition, the soft tissue component of MCS was also detected in the US image. Selecting the cases properly in the US would help determine the characteristic features of malignant conditions involving the bone, even before any access to histopathologic results of incisional biopsy [10].

Our case presented a mixed radiolucent–radiopaque mass lesion located at the right mandibular ramus from the second molar to the sigmoid notch and from the alveolar crest to the inferior border of the mandible. It has invasive behavior, such as irregular osseous formation causing internal sunray appearance alongside Codman triangle periosteal reaction and PDL widening of the second molar. The lesion has also perforated the buccal and lingual cortex and extended to the soft tissue, which favors malignancy.

Due to the patient's age (38 years old) and a higher rate of occurrence, osteosarcoma should be considered a differential diagnosis. Chondrosarcoma is the second choice due to its older mean age of occurrence and rarity. Osteogenic metastases such as prostate or breast cancer and other sarcomas can also be suggested as other possible pathologies.

Bueno et al. reported a case of MCS in a 28-year-old female patient, which mimicked apical periodontitis. The patient underwent root canal treatment. However, after 45 days, she experienced swelling, intense pain, and a suspected furcation lesion, leading to the extraction of the tooth. Rapid swelling occurred days after the extraction, which prompted a referral to an oral and maxillofacial surgeon and resulted in the final diagnosis. In such cases, careful evaluation and precise differential diagnosis are essential, especially for those with expansion on buccal and lingual plates [33].

Histologically, MCS is characterized by a biphasic pattern of areas of hyaline cartilage mixed with small round cell proliferation [25, 29]. It has proven challenging to diagnose on incisional biopsy [2].

When there is an abundance of cartilage in MCS, it is easily visible in random tumor sections. This may make the diagnosis much more effortless. This cartilage is the interface between the stromal round cells, and its appearance is usually quite distinctive. When the cartilaginous component is absent in small biopsy specimens, various small-cell neoplasms must be considered for differential diagnosis. These may encompass lymphoma, Ewing sarcoma, angiosarcomas, hemangiopericytoma, synovial sarcoma, melanoma, undifferentiated carcinoma, small-cell osteosarcoma, chondroblastic osteosarcomas, MCS, embryonal rhabdomyosarcoma, and neuroblastoma [11].

It is important to note that immunohistochemical investigations and molecular diagnostic techniques, specifically in conjunction with paraffin-embedded tissue samples, may yield diagnostically valuable information.

Both Ewing sarcoma and MCS show similar histological findings that can exhibit a plentiful, thin-walled vascular network among the tumoral cells and staghorn spaces, resembling hemangiopericytoma, as seen in our current case. Ewing sarcoma is positive for vimentin and CD99 but lacks chondroid areas. Though well-differentiated cartilage has been reported in one case of benign hemangiopericytoma, it is a rare finding [12]. Lee et al. showed that, in contrast to Ewing sarcoma, small-cell osteosarcoma and MCS lack FLI-1 immunoreactivity. FLI-1 is, therefore, useful in the differential diagnosis of small round blue cell tumors of the bone [19, 46].

In our case, we faced a small round-cell tumor without any calcified material in the incisional biopsy.

We ruled out undifferentiated carcinoma, lymphoma, melanoma, and embryonal rhabdomyosarcoma based on negative immunoreactions of PanCK, LCA, HMB45, and MyoD1, respectively. However, we could not rule out Ewing sarcoma or bone/cartilage-forming tumors. Based on the fact that the surgical treatment is the first treatment plan for all of these sarcomas, the surgeon decided to excise the whole lesion with safe margins after consultation in the hospital tumor board discussion meetings.

Several hundred specific gene fusions have been identified in human cancers, and many of these have emerged as diagnostic or prognostic markers, and some as therapeutic targets. Characteristic genetic changes in many sarcoma types, a majority of which were specific gene fusions, have been reported. Indeed, recurrent, tumor type–specific gene fusions have been identified in many histological subtypes of sarcomas [47].

In 2012, *HEY1-NCOA2* fusion was the first recurrent gene fusion identified in MCSs [47]. Subsequently, IRF2BP2*-*CDX1 fusion was reported [48]. In 2020, NKX3.1 expression was reported as a useful immunohistochemical marker for MCS [49, 50].

Another well-known diagnostic marker in MCS is the lack of isocitrate dehydrogenase (*IDH*)1/2 mutations, common in central, periosteal, and dedifferentiated CS [51, 52]. By identifying such fusion genes or confirming NKX3.1 expression by IHC, it is easier to accurately diagnose MCS [47, 51].

Hakeem et al. reported a case of a patient who received an initial diagnosis of Ewing sarcoma due to limited tissue for biopsy. This led to unnecessary neoadjuvant chemotherapy. After no response, a second opinion recommended obtaining a representative pathology specimen and consulting an experienced pathologist to ensure accurate diagnosis and proper treatment. They used the HEY1-NCOA2 fusion transcript as a potential diagnostic tool identified for MCSs to help differentiate it from other variants [17].

Regarding tumorigenesis, Kishikawa et al. and Qi et al. reported that platelet-derived growth factor receptor alpha, which belongs to a family of receptor tyrosine kinases, was upregulated by *HEY1-NCOA2* fusion in a study. In addition, a recent report showed that imatinib, a tyrosine kinase inhibitor (TKI), significantly reduced tumor growth in the *HEY1-NCOA2* fusion-driven cellular model as well as in MCS patient–derived xenograft models. Therefore, further research is required, and TKI may be effective in treating MCS [53, 54].

Singh et al. reported a case of MCHS of the mandible that arose in a 22-year-old woman whose initial two biopsies did not reveal the features of the tumor. The first diagnosis was made after FNAC identified giant-cell reparative granuloma, and the patient was prescribed an antibiotic and a steroid. As the patient did not experience any relief after 6 weeks, an incisional biopsy was performed, which indicated that the lesion was a benign calcifying epithelial odontogenic tumor. Incision and curettage of the tumor were performed. However, 6 months after the patient's initial presentation, the mandibular swelling had receded and increased in size. Further investigations revealed MCS [26].

Furthermore, the cartilage in MCS may display a spectrum ranging from normal cartilage to low-grade chondrosarcoma. Conversely, high-grade malignant cartilage-forming tumors typically occur in small-cell osteosarcoma.

Differentiating chondroblastic osteosarcomas may not be difficult as they show malignant spindle cells toward the periphery of the lobules with the presence of lace-like tumor osteoid. In contrast, MCS lacks the tumor osteoid [12].

MCS shows clustering of intratumoral blood vessels, which may have to be distinguished from angiosarcomas. However, these lesions do not demonstrate chondroid components such as those of MCS and are positive for vascular markers CD31 and CD34.

Synovial sarcoma and some spindle-cell variants share similar histology, but S100 positivity and CK negativity will enable the demarcation of these tumors. Metaplastic cartilage may also occur in poorly differentiated synovial sarcoma, but it is much less common than foci of calcification or bone. In complex cases, a careful search for a biphasic pattern or epithelial differentiation with antibodies against cytokeratin or epithelial membrane antigen is necessary [12].

Nevertheless, in cases like ours, a major challenge arises when cartilage is scarce, necessitating multiple sections for its detection in incisional biopsies and subserial sectioning.

En bloc resection with wide margins (2–3 cm) resulting in a lower rate of reoccurrence is usually recommended as the treatment procedure unless anatomic consideration, especially for tumors in the axial regions, alters the decision and a better survival rate than limited surgical resection [4, 8, 25, 55]. Due to a strong tendency of MCS toward local recurrence and distant metastases many years after primary treatment, the necessity of long-term surveillance should be emphasized. Compared to other variants of chondrosarcomas, MCS has shown more sensitivity to chemotherapy and radiation [56]. Negative surgical margins prove to be significantly associated with improved survival rates. Without clear surgical margins, RT should be implemented as a salvage therapy. According to several studies, perioperative chemotherapy may reduce local recurrence, but its possible survival benefit remains unclear. In the case of distant metastases, treatment should be determined individually based on the patients' age and general state of health. Despite the limited efficacy of conventional chemotherapy in an advanced setting, young patients may be considered for chemotherapy combined with aggressive local treatment with surgery and/or radiotherapy. In contrast, elderly patients should be treated with palliative intent [52]. After successful surgical therapy with inconspicuous resection margins, the 5-year survival rate is still only 50% because of a tendency to recur locally uncontrollably. Prognosis is generally poorer in the head and neck region than in more frequently involved body parts [45].

In the presented case, a resection with wide free margins was performed. Mesh prostheses were used to reconstruct the mandible and temporomandibular joints to regain function and cosmetics. Our patient had five chemotherapy and 25 radiotherapy sessions after tumor removal.

## 4. Conclusion

In conclusion, an incisional biopsy frequently fails to provide sufficient tissue to establish an MCS diagnosis. Adequate tissue with multiple sections, detailed histopathological examination, and adjunctive IHC are the keys to a definitive diagnosis. Long-term follow-up should be considered due to the high recurrence rate and possible distant metastasis often occurring with MCS. This case provides valuable and informative data and insights, contributing to our understanding of this rare entity with limited reported cases.

## Figures and Tables

**Table 1:** Reported cases of mandibular mesenchymal chondrosarcoma.

**Reported case (author/year of publication)**	**Demographics (age/gender)**	**Location**	**Treatment**	**Follow-up**
Dang et al., 2023 [15]	42/M	Man/R/condyle	Resection Chemotherapy	Metastasis to the lung after 6 months. After 8 years of follow-up, there were metastases to the pancreas
Chidzonga et al., 2023 [16]	29/F	Man/L	Resection	No data available
Hakeem et al., 2023 [17]	11/M	Man/L	Resection and neck dissection Radiotherapy	3-year follow-up No recurrence
Dani, 2022 [18]	34/F	Man/Ant	Excision	2-year follow-up No evidence of recurrence
Derakhshan et al., 2021 [19]	19/M	Man/Ant	Partial mandibulectomy Radiotherapy	After 14 months, the patient died of several distant metastases
Abdelmalak, 2020 [20]	25/F	Man/L	Mandibular segmental resection Chemotherapy	No data available
Almansoori et al., 2019 [21]	39/F	Man/L	Hemimandibulectomy	5-year follow-up Recurrence and lung metastasis
George et al., 2019 [22]	16/M	Man/Ant	Resection Chemotherapy	3-year follow-up No recurrence No metastasis
Abouchadi et al., 2018 [23]	73/F	Man/R	Hemimandibulectomy Cervical lymph node dissection	No recurrences
Suwal et al., 2017 [24]	54/F	Man/L	Resection of the mandible with left total neck dissection	6-month follow-up No obvious complications
Majumdar et al., 2016 [12]	60/F	Man/L	Surgical excision	No metastasis
Singh et al., 2014 [25]	60/M	Man/R	Surgical resection	2-year follow-up No recurrence
Singh et al., 2014 [26]	22/F	Man/R	FNAC identified a giant-cell reparative granuloma, and the patient was prescribed an antibiotic and a steroid Hemimandibulectomy Right submandibular gland excision and neck dissection	No data available
Shakked et al., 2012 [27]	31/M	Man	Resection Chemotherapy	12.5-year follow-up
Shakked et al., 2012 [27]	35/F	Man	Resection	12-year follow-up
Shakked et al., 2012 [27]	27/M	Man	Resection	Expired after 4.5-year follow-up
Ram et al., 2011 [8]	19/M	Man/Ant	Resection	Recurrence after a year and excision as treatment
Shahidi et al., 2011 [10]	24/M	Man/L	Resection	The patient did not return for follow-up
Krishnamurthy et al., 2011 [28]	34/M	Man/L	Radiotherapy Chemotherapy	The patient died 3 months after diagnosis
Cheim et al., 2011 [29]	31/F	Man/R	Resection	3-Year follow-up No lymph node involvement No recurrence
Suh et al., 2010 [30]	52/F	Man/R	Resection	
Reeby et al., 2010 [31]	22/M	Man/R	Hemimandibulectomy	No data available
Myers, Jiang, and Girotto, 2009 [32]	19/M	Man/R	Resection	2-year follow up No metastasis No recurrence
Bueno et al., 2008 [33]	16/F	Man	Not mentioned	Showed single metastasis after 26-year follow-up
Kumaraswamy Naik et al., 2008 [34]	23/F	Man/L	Resection	The patient did not return for a follow-up. She had expired after 6 months due to lung metastasis
Uchiyama et al., 2008 [35]	33/M	Man/R	Resection	3-year follow-up Recurrence Multiple pulmonary metastases after 10 months of diagnosed recurrence
Kumaraswamy Naik et al., 2008 [34]	23/F	Man/L	Resection	The patient died of lung metastasis after 6 months of diagnosis
Angiero et al., 2007 [36]	64/F	Man/L	Hemimandibulectomy	8-year follow-up No recurrence
Bencheikh et al., 2007 [37]	23/M	Man/R	Hemimandibulectomy	6-month follow-up No recurrence
Al-Bayaty et al., 2003 [38]	18/F	Man/R	Partial mandibulectomy	The patient expired after an 18-month follow-up
Huffer, Ketch, and Greer, 1999 [39]	27/F	Man/L	Hemimandibulectomy	No data available
Zakkak et al., 1998 [40]	31/M	Man/Ant	Resection No chemotherapy or radiation treatment	1-year follow-up No metastasis No recurrence
Vêncio et al., 1998 [41]	23/M	Man	Hemimandibulectomy Chemotherapy radiation	4-year follow-up Metastasis to lower back The patient expired
Vêncio et al., 1998 [41]	51/M	Man	Hemimandibulectomy Chemotherapy radiation	11-month follow up No metastasis No recurrence
Vêncio et al., 1998 [41]	12/M	Man	Hemimandibulectomy	11-year follow-up No metastasis No recurrence
Vêncio et al., 1998 [41]	8/M	Man	Hemimandibulectomy	31-year follow-up Recurrence after 10 years
Vêncio et al., 1998 [41]	11/M	Man	Hemimandibulectomy	No follow-up
Vêncio et al., 1998 [41]	2/M	Man	Resection Chemotherapy	8-year follow-up No metastasis Recurrence
Vêncio et al., 1998 [41]	23/F	Man	Resection chemotherapy	2-year follow-up No metastasis Recurrence
Vêncio et al., 1998 [41]	16/F	Man	Hemimandibulectomy	22-year follow-up Metastasis to the scalp
Vêncio et al., 1998 [41]	12/F	Man	Hemimandibulectomy	6-year follow-up No evidence of disease or metastasis
Vêncio et al., 1998 [41]	21/F	Man	No data available	No data available
Hollins et al., 1987 [42]	27/M	Man/Ant	Resection Chemotherapy Radiotherapy	The patient expired 72 months after the initial diagnosis
Caravolas et al., 1981 [43]	12/M	Man/L	Resection	No data available
Harwood, Krajbich, and Fornasier, 1980 [44]	70/F	Man	Radiotherapy	The patient expired after a year

Abbreviations: Ant, anterior; F, female; L, left; M, male; Man, mandible; R, right.

## Data Availability

The authors confirm that the data supporting the findings of this study are available within the article.
